# Portal annular pancreas in chronic pancreatitis: technical challenges in surgical management

**DOI:** 10.3389/fsurg.2026.1982811

**Published:** 2026-10-26

**Authors:** Aparna Jagannathan, Anoop Paul, Siddharth M. Babu, Arpana Lily Jessica, Joseph Chacko Paul, Ajith Thomas, Ravish Sanghi Raju, Philip Joseph

**Affiliations:** 1 Department of Hepatic Pancreatic and Biliary Surgery, Christian Medical College Vellore, Vellore, India; 2 Department of Radiology, Christian Medical College Vellore, Vellore, India; 3 Department of Gastroenterology, Christian Medical College Vellore, Vellore, India

**Keywords:** chronic pancreatitis, circumportal annular pancreas, Frey’s procedure, portal annular pancreas, retroportal main pancreatic duct

## Abstract

**Introduction:**

Portal annular pancreas (PAP) is a rare anatomical variant characterized by abnormal fusion of pancreatic tissue between the uncinate process and the body of the pancreas encircling the portal vein. Although the technique of resection in pancreaticoduodenectomy has been described previously, in chronic pancreatitis, drainage procedures may also be required, rendering access to the retroportal segment of the portal annular pancreas technically challenging. In this study, we outline various management strategies based on the type of PAP and the involvement of the retroportal pancreatic duct.

**Methods:**

This is a retrospective review of all patients diagnosed with chronic pancreatitis and PAP between January 2006 and September 2025. Information on patient demographics, the type of PAP, and treatment approaches was collected from electronic medical records and also by reviewing the cross-sectional imaging results of patients.

**Results:**

Five patients were identified with both chronic pancreatitis and PAP and were classified under three categories: one with type 1, one with type 2, and three with type 3 PAP. Three patients underwent surgery: one patient presented with obstructive jaundice and therefore underwent an extended pancreaticoduodenectomy beyond the splenic artery to excise the retroportal pancreas; one patient underwent a Frey's procedure, as the duct of the retroportal pancreas was not involved; and one patient required a duodenum-preserving extended resection, including subtotal excision of the head of pancreas with PAP and pancreatojejunostomy, due to the retroportal course of the main pancreatic duct.

**Conclusions:**

This study presents the first reported single-center limited case series describing the management of chronic pancreatitis associated with PAP. A tailored surgical approach was shown to be safe and effective, resulting in minimal postoperative morbidity and satisfactory pain relief.

## Introduction

Portal annular pancreas (PAP), also referred to as circumportal pancreas (1) or complete pancreatic encasement, is a rare congenital anomaly in which the uncinate process of the pancreas encircles the portal vein and/or the superior mesenteric/splenic vein, eventually fusing with the dorsal aspect of the pancreatic body. The prevalence of PAP ranges from 1.1% to 3.4%, which is determined based on prior computed tomography (CT) imaging studies in healthy individuals or incidental findings in patients (2, 3).

The classification of PAP is based on the anatomical course of the main pancreatic duct (MPD) in relation to the portal venous confluence. Our classification (2009) (4) of PAP is given as follows:

- Type 1 PAP involves fusion of the ventral pancreas with the body and a retroportal main pancreatic duct (RMPD).
- Type 2 PAP is similar to type 1, but it occurs in the presence of pancreas divisum.
- Type 3 PAP features encasement of the portal vein by the uncinate process with a normally positioned anteportal MPD (AMPD).

Each type is further classified into A, B, and C based on its anatomical relationship to the superior mesenteric vein–portal vein confluence, whether it is suprasplenic, infrasplenic, or mixed, respectively (5).

Chronic pancreatitis is a significant cause of long-term morbidity and can result from both parenchymal and ductal abnormalities (6). Common etiological factors include chronic alcohol use, trauma, genetic predisposition, and structural anomalies such as pancreas divisum or anomalous pancreaticobiliary duct junctions (APBDJs) (7–10). In addition, the “meandering main pancreatic duct” (MMPD), which is an abnormal ductal course in the ventral head, has been proposed as a potential contributor to idiopathic forms of pancreatitis (11).

The aim of surgical management in chronic pancreatitis is pain relief and reduction of complications. The options are drainage procedures, resection procedures, and hybrid operations (12). Although the strategy for resection procedures (pancreaticoduodenectomy) for PAP will remain the same as described for malignancies, drainage procedures such as Partington–Rochelle modification of the Puestow–Gillesby procedure (13) or hybrid procedures such as Frey, Beger, or Imaizumi procedures (14–16) are complicated by the lack of access to the retroportal portion (Figure 1). Therefore, there is a need to develop an algorithm to address this retroportal portion.

To the best of our knowledge, there has been no study on the clinical relevance of PAP in the management of chronic pancreatitis.

**Figure 1:**
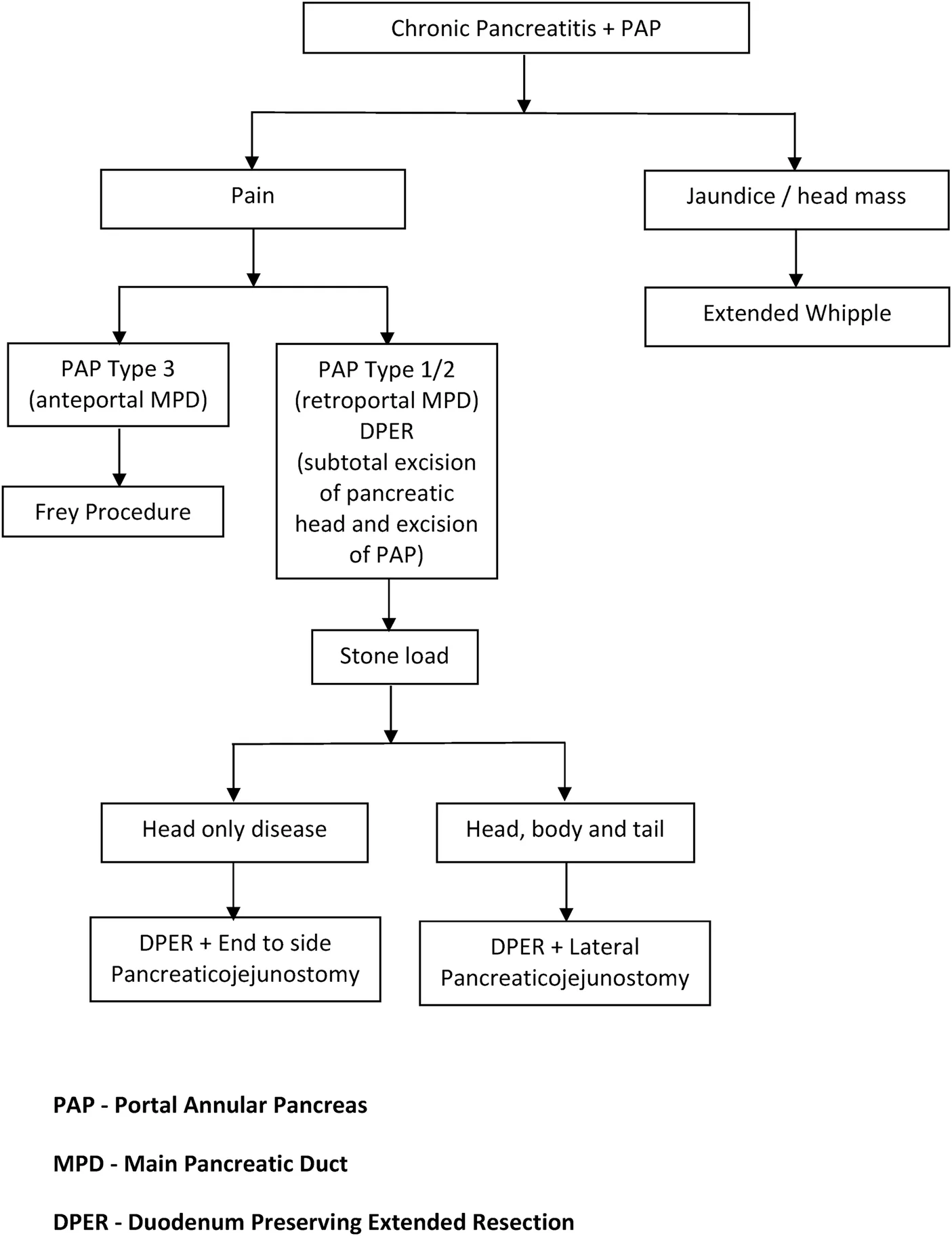
A PAP management algorithm.

## Material and methods

This is a retrospective observational study. All patients who were diagnosed with both chronic pancreatitis and PAP between January 2006 and September 2025 were included. The electronic medical records were reviewed, and the cross-sectional imaging results of these patients were reviewed on Picture Archiving and Communication System (PACS).

Two abdominal radiology consultants independently reviewed the CT and MRI results for each case. A diagnosis of PAP was confirmed when pancreatic tissue was seen completely encircling the portal vein or the superior mesenteric vein on at least two consecutive axial slices. The following features were independently assessed by two radiologists, and there was 100% concordance between them for all four criteria.

1. The course of the main pancreatic duct in relation to the portal vein (classified as anteportal or retroportal)
2. The location of the encirclement of the portal vein by the pancreatic tissue in relation to the splenic vein (classified as suprasplenic, infrasplenic, or mixed)
3. The presence of an accessory pancreatic duct posterior to the portal vein
4. Radiological features of chronic pancreatitis

The patients were further categorized according to the classification system previously described at our institution based on the ductal course and the site of anomalous pancreatic fusion. Intraoperative findings and surgical records were reviewed for confirmation. All surgical procedures were performed following multidisciplinary team (MDT) discussions, and modifications to standard techniques were made in line with the existing departmental protocols.

## Results

A total of five patients (*N* = 5) diagnosed with chronic pancreatitis and portal annular pancreas were included in the final analysis: one with type 1, one with type 2, and three with type 3 PAP (Table 1).

**Table 1:** Patient demographics.

Patient no.	Age (at presentation)/sex	Etiology	Pain	Jaundice	Exocrine insufficiency	Endocrine insufficiency	Type of PAP	Distribution of calculi	Trial of non-operative medication
1	34/M	Idiopathic	+	−	+	−	3C (Figures 2, 3)	Head, tail, anteportal MPD	+
2	37/M	Ethanol	+	−	+	+	3A (Figures 4, 5)	Head, body, tail, anteportal MPD	+
3	39/M	Idiopathic	+	−	+	+	1C (Figures 6, 7)	No stones, only duct changes, retroportal MPD	+
4	33/F	Idiopathic	+	+	+	+	3A (Figures 8–11)	Head, anteportal MPD	−
5	21/F	Idiopathic	+	−	+	−	2A (Figures 12, 13)	Head, body, tail, retroportal MPD	+

All patients presented with upper abdominal pain. One of these patients also had obstructive jaundice. Contrast-enhanced CT imaging and Magnetic Resonance Cholangiopancreatography (MRCP) were performed in all patients. In three patients (patients 1,2 and 4), the main pancreatic duct had an anteportal course, whereas in the other two patients, it had a retroportal course (patients 3 and 5). In all the patients, the uncinate process was fused to the dorsal aspect of the pancreatic body. The line of fusion in relation to the splenic vein was suprasplenic in three patients, infrasplenic in none, and mixed in two.

Management was based on the presenting symptoms and the type of portal annular pancreas (Table 2). One patient achieved good symptomatic relief via medical management (Patient 2) and did not undergo surgery. Another patient did not follow up (Patient 3). Three patients underwent surgery: two of them had persistent abdominal pain after a medical management trial and one presented with obstructive jaundice. The one with jaundice (Patient 4) underwent an extended pancreaticoduodenectomy, which included resection beyond the splenic artery at the line of fusion of the retroportal pancreas with the dorsal pancreas. The histopathology results did not show any malignancy, and the jaundice was probably secondary to the distal biliary stricture.

**Table 2:** Treatment and follow-up.

Patient no.	Operation done	Postoperative morbidity (Clavien–Dindo grade)	Hospital stay	Follow-up (months)	Pain relief (VAS score)
1	Frey’s procedure	Grade 1	10	20	Adequate (3)
2	—	—	—	—	Only on medical management (5)
3	—	—	—	—	Lost to follow-up
4	Extended PD (PD + PAP excision)	Grade 2	14	18	Adequate (2)
5	DPER (duodenum-preserving extended resection)–subtotal excision of the head of pancreas and PAP with pancreatojejunostomy	Grade 2	14	11	Adequate (3)

Among the two patients who experienced pain, one (patient 1) underwent Frey's procedure because the retroportal annular process was not involved (Figures 2, 3). The other patient (patient 5) had a retroportal course of MPD (Figures 12, 13) and required a duodenum-preserving extended resection (DPER), which involved a subtotal resection of the head of the pancreas and excision of PAP after the creation of the secondary tunnel (supramesenteric–coeliac arterial tunnel). This extended resection was beyond the splenic artery at the line of fusion with the dorsal pancreas (Figures 17–19).

**Figure 2:**
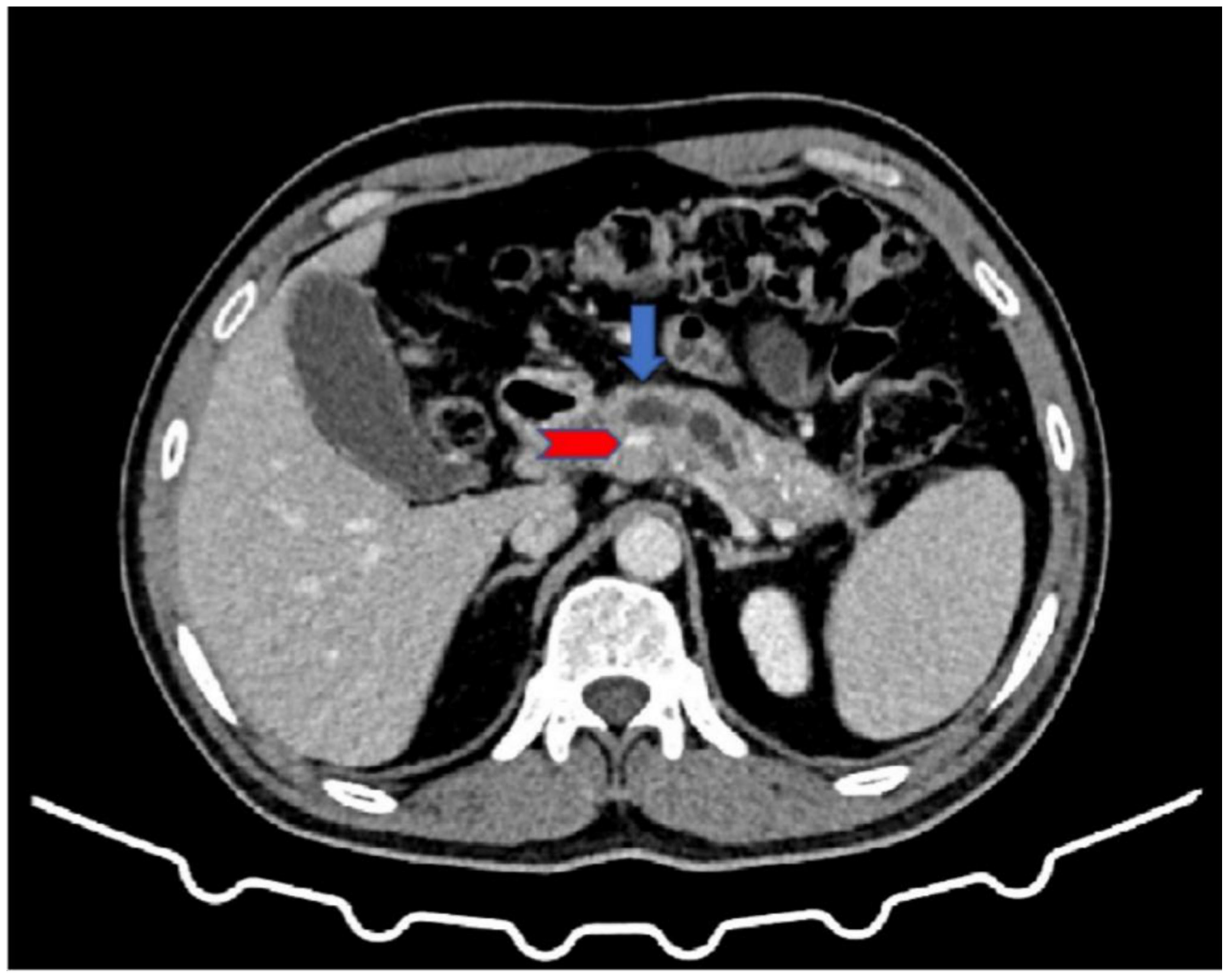
Patient 1: an abdominal CT scan shows features of chronic calcific pancreatitis with a portal annular pancreas. Dilated MPD (blue block arrow) runs anteriorly to the portal vein (indicated by the red chevron arrow).

**Figure 3:**
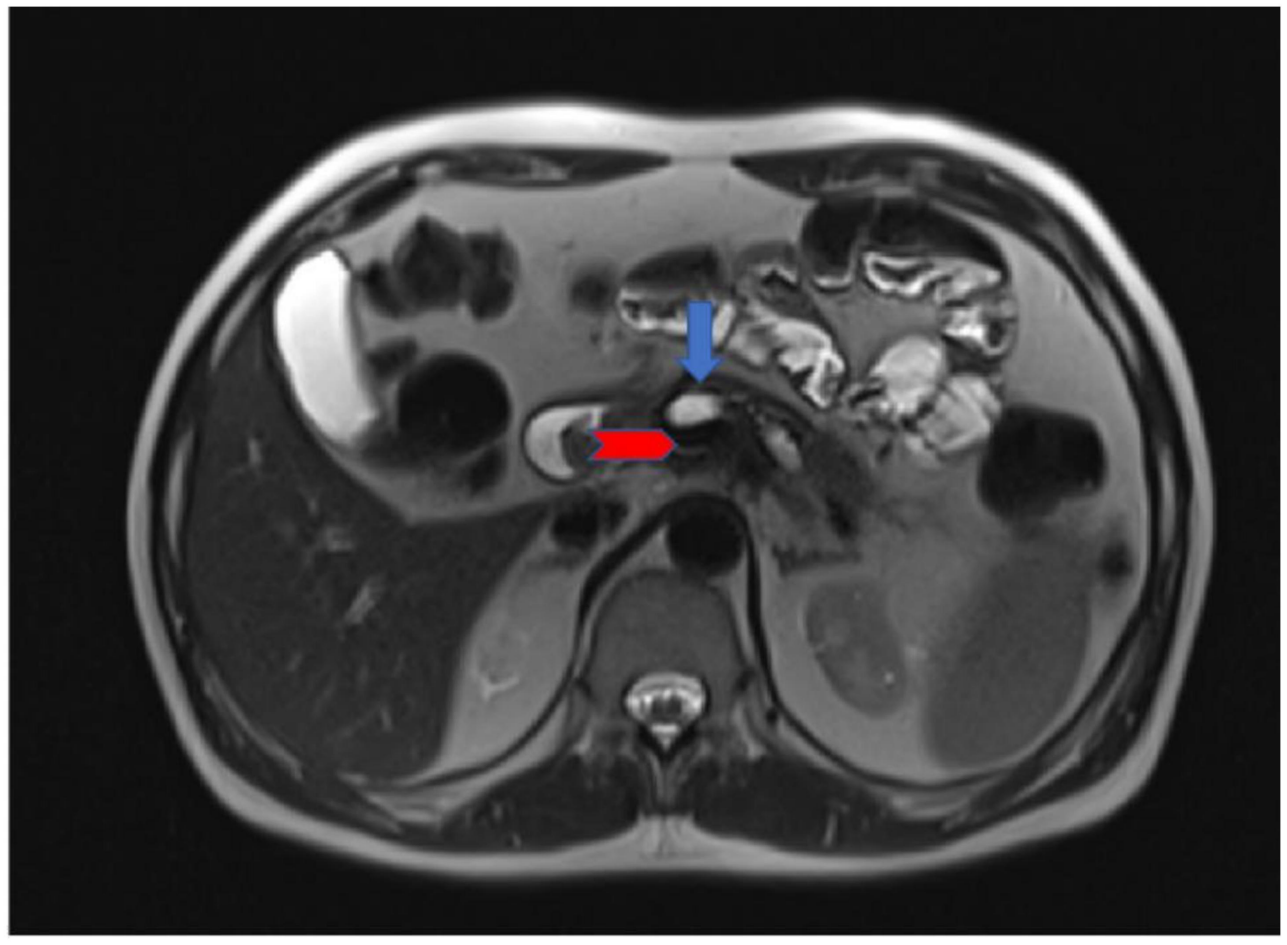
Patient 1: MRCP shows features of chronic calcific pancreatitis with a portal annular pancreas. Dilated MPD (indicated by the blue block arrow) runs anteriorly to the portal vein (indicated by the red chevron arrow).

**Figure 4:**
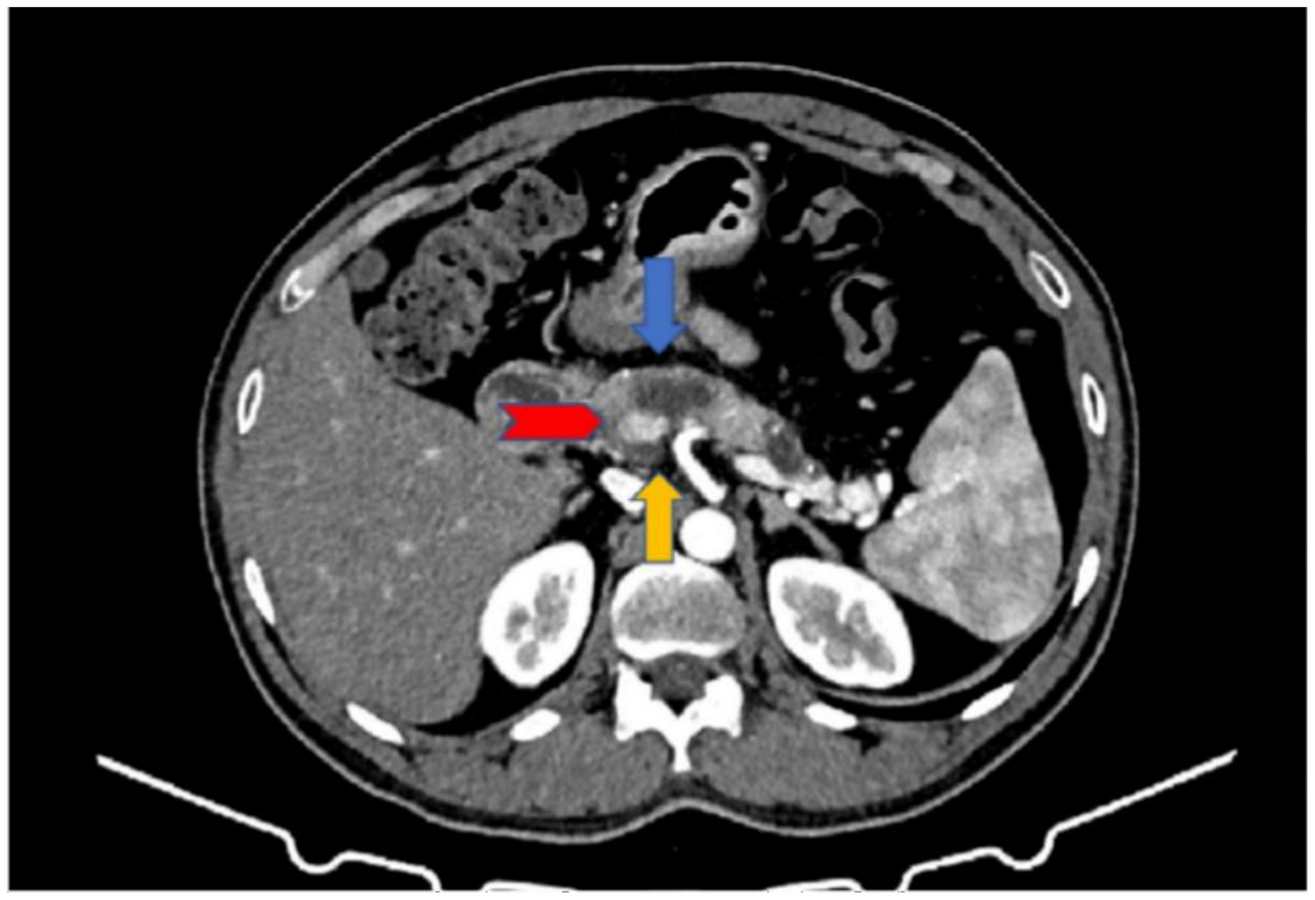
Patient 2: an abdominal CT scan shows features of chronic calcific pancreatitis with a portal annular pancreas. Dilated MPD (indicated by the blue block arrow) runs anteriorly to the portal vein (indicated by the red chevron arrow). An accessory pancreatic duct (indicated by the yellow block arrow) is noted coursing posteriorly to the portal vein.

**Figure 5:**
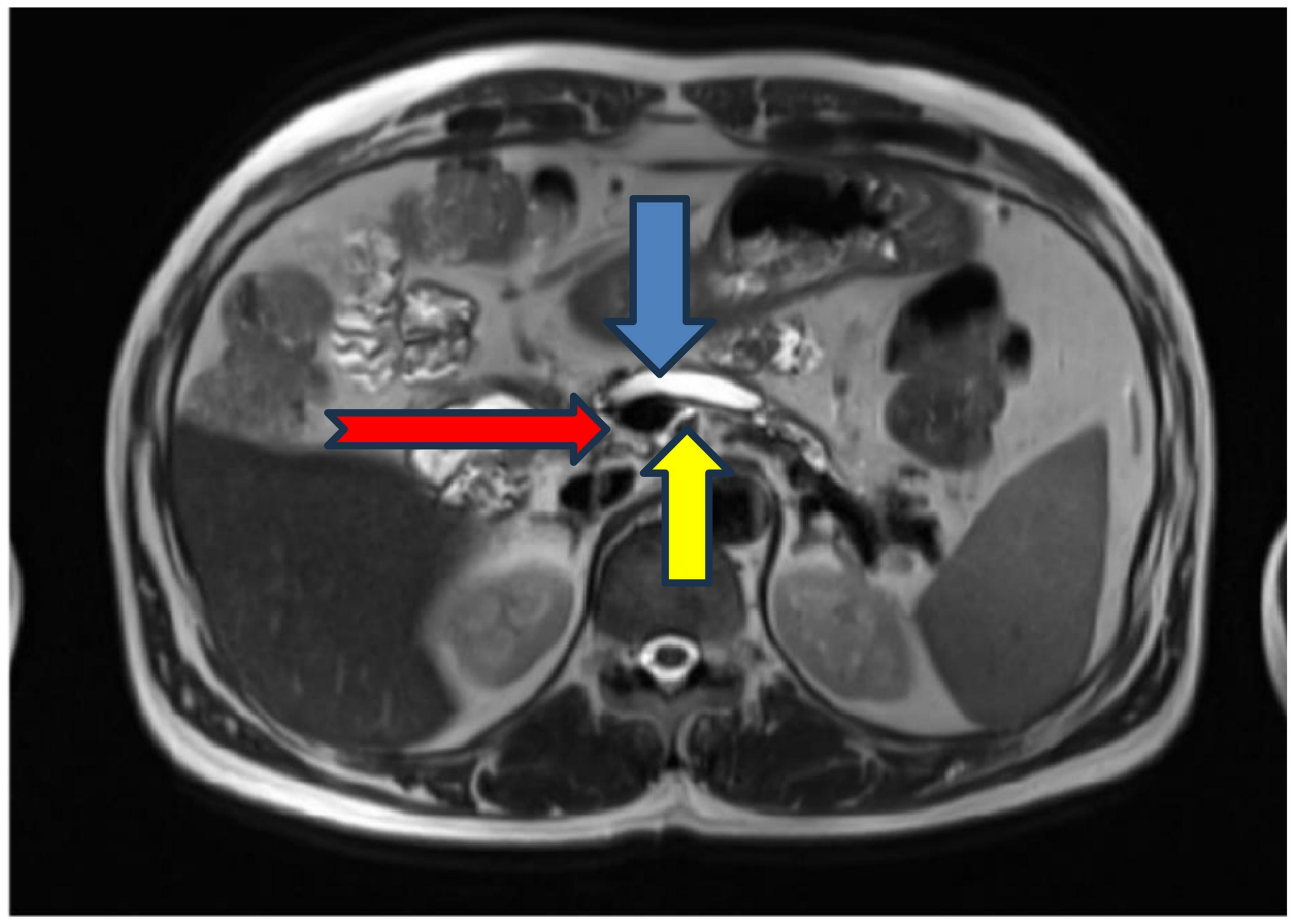
Patient 2: MRCP shows features of chronic calcific pancreatitis with a portal annular pancreas. Dilated MPD (indicated by the blue block arrow) runs anteriorly to the portal vein (indicated by the red chevron arrow). An accessory pancreatic duct is noted coursing posteriorly to the portal vein (indicated by the yellow block arrow).

**Figure 6:**
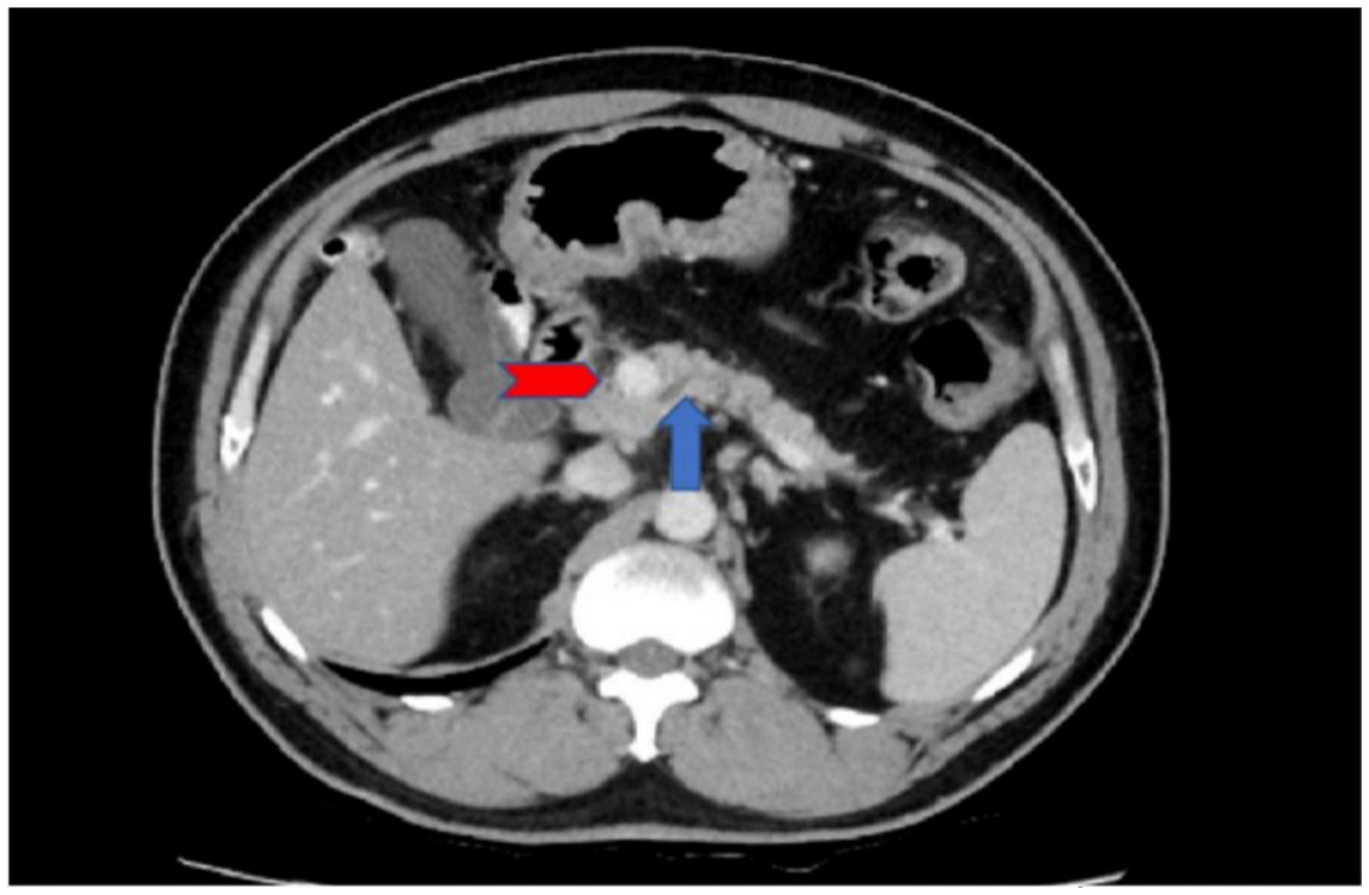
Patient 3: an abdominal CT scan shows features of chronic pancreatitis with a portal annular pancreas. MPD (indicated by the blue block arrow) runs posteriorly to the portal vein (indicated by the red chevron arrow).

**Figure 7:**
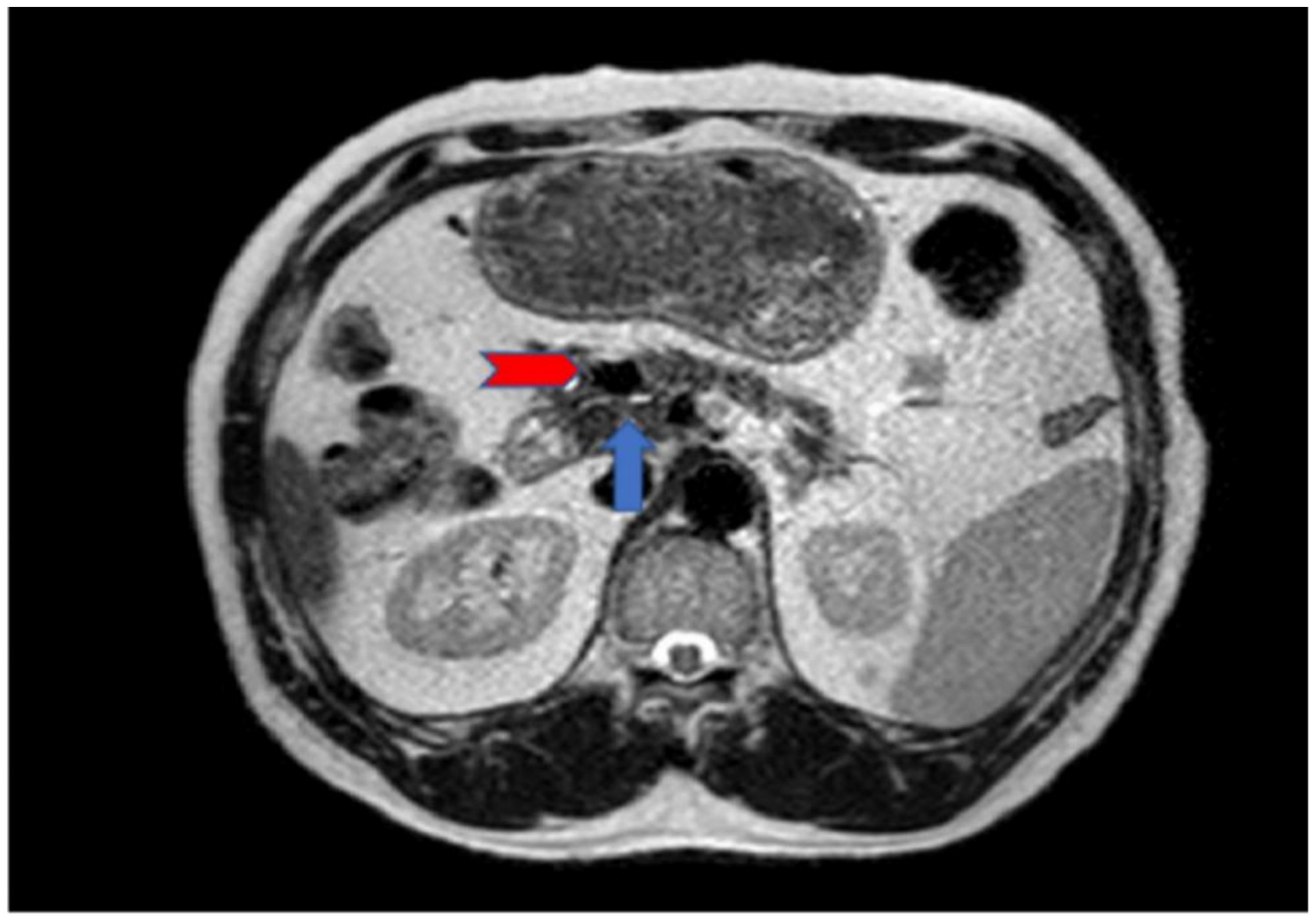
Patient 3: MRCP shows features of chronic pancreatitis with a portal annular pancreas. MPD (indicated by the blue block arrow) runs posteriorly to the portal vein (indicated by the red chevron arrow).

**Figure 8:**
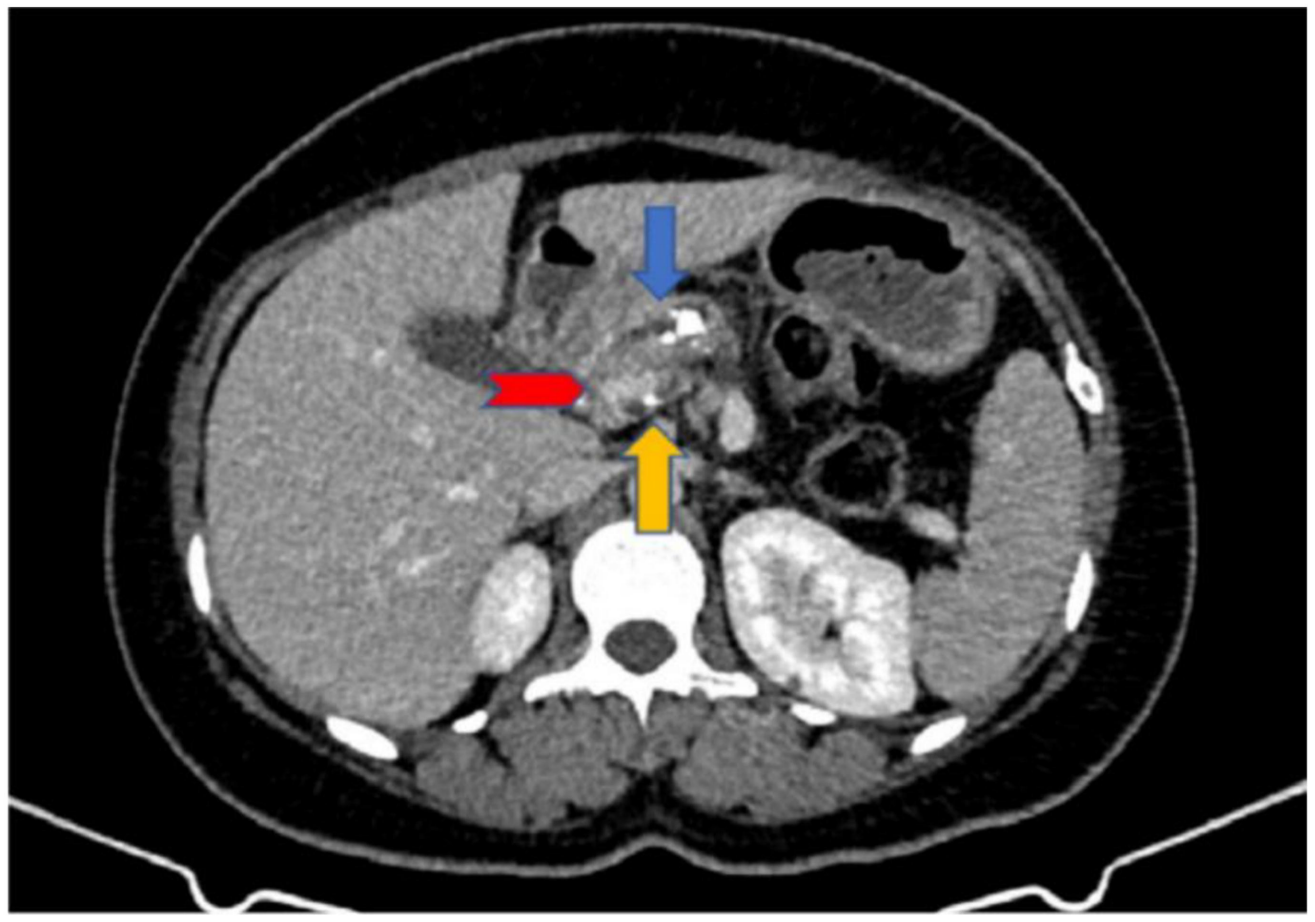
Patient 4: An abdominal CT scan shows features of chronic calcific pancreatitis with a portal annular pancreas. Dilated MPD (indicated by the blue block arrow) runs anteriorly to the portal vein (indicated by the red chevron arrow). An accessory pancreatic duct (indicated by the yellow block arrow) is noted coursing posteriorly to the portal vein.

**Figure 9:**
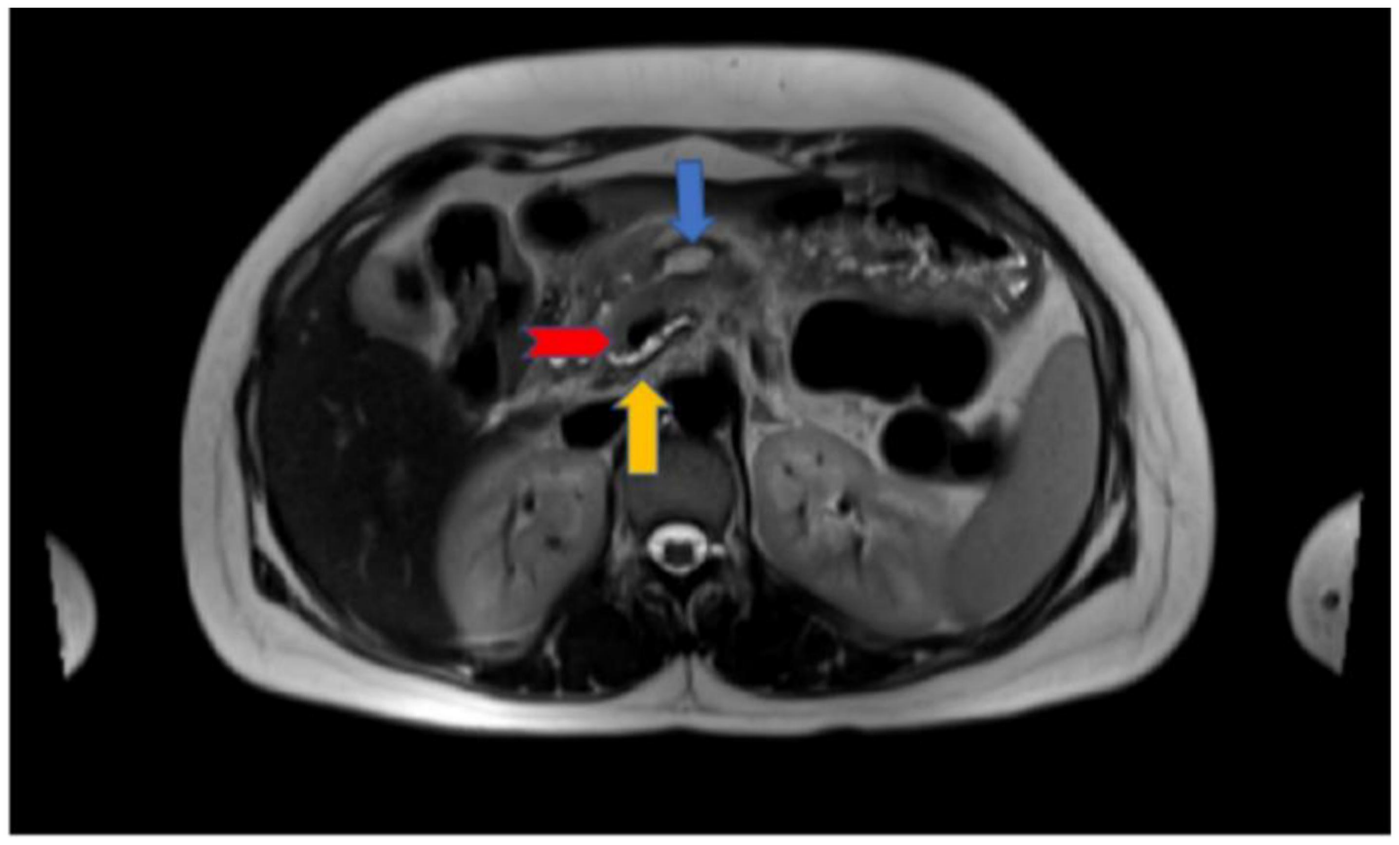
Patient 4: MRCP shows features of chronic calcific pancreatitis with a portal annular pancreas. Dilated MPD (indicated by the blue block arrow) runs anterior to the portal vein (indicated by the red chevron arrow). An accessory pancreatic duct (indicated by the yellow block arrow) is noted coursing posteriorly to the portal vein.

**Figure 10:**
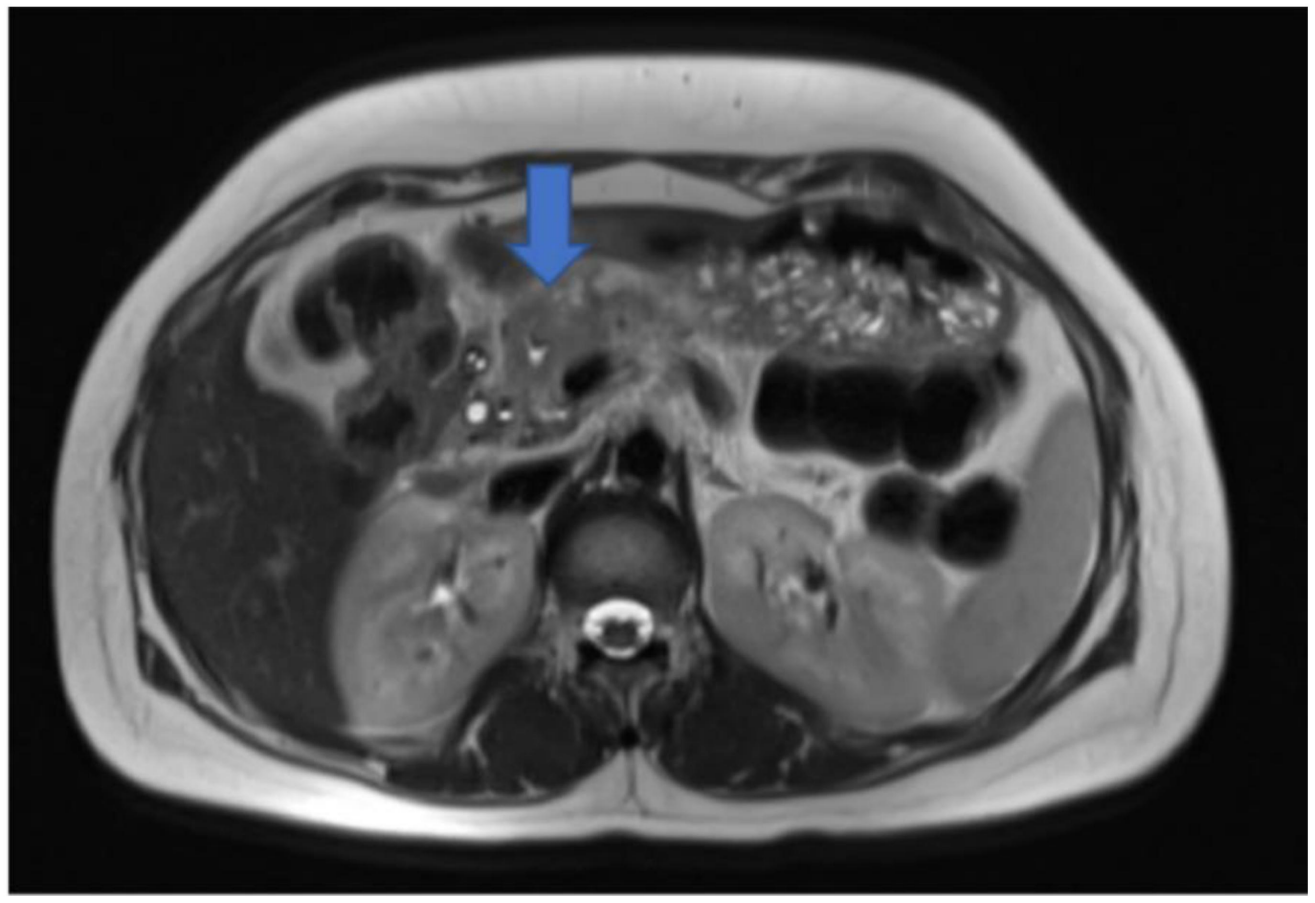
Patient 4: MRCP shows an enlarged pancreatic head (indicated by the blue arrow).

**Figure 11:**
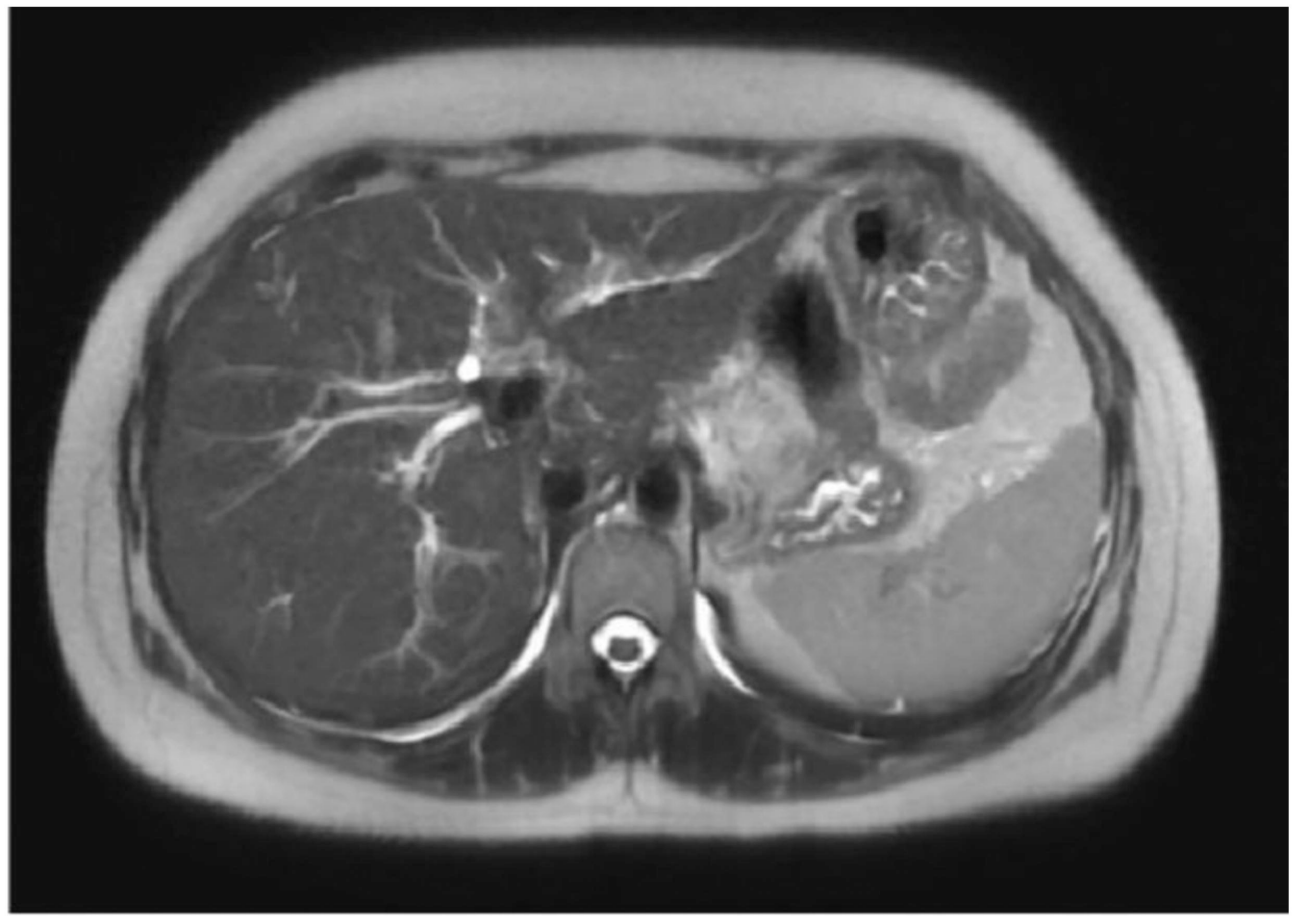
Patient 4: MRCP shows upstream intrahepatic biliary radicle dilatation.

**Figure 12:**
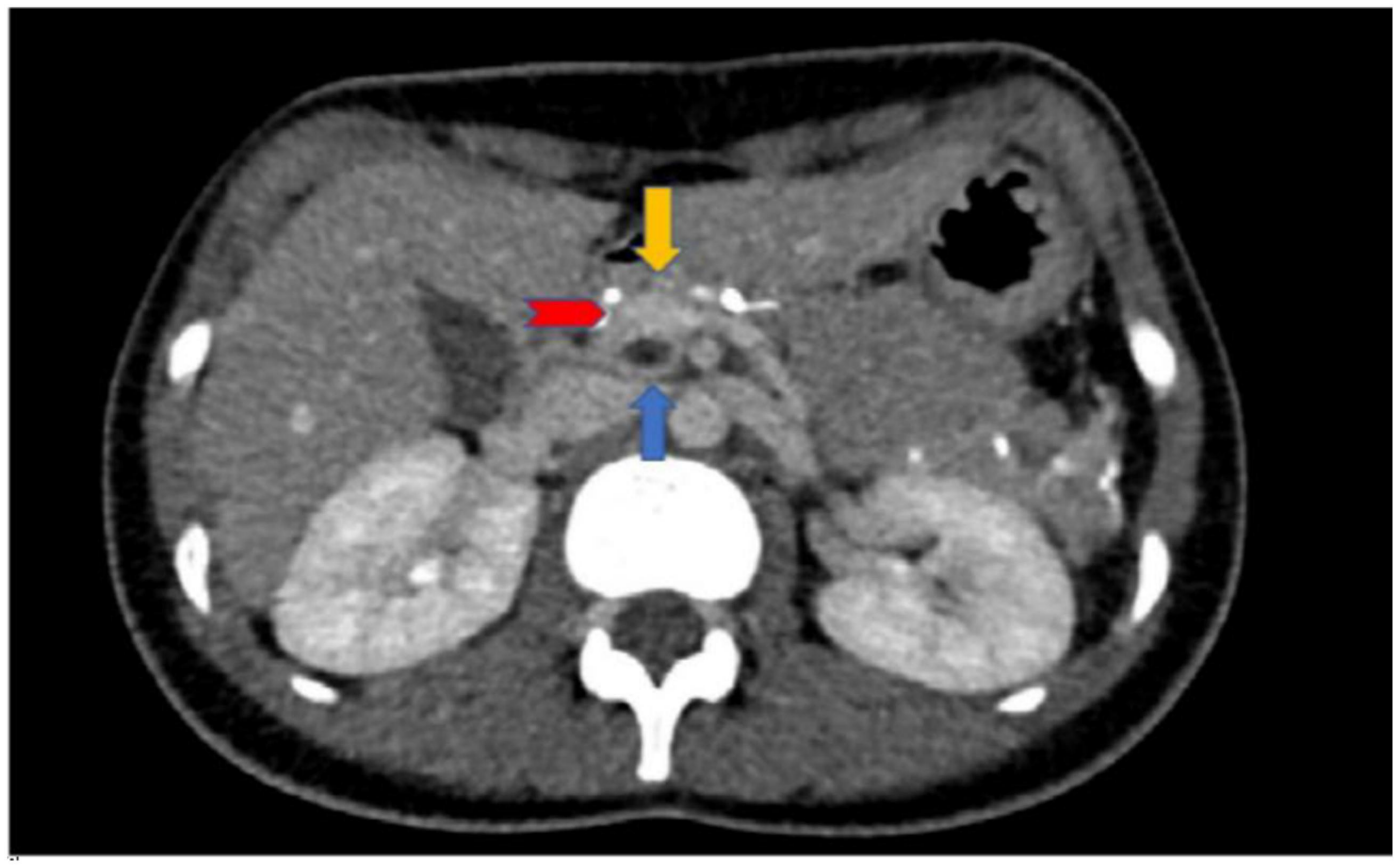
Patient 5: an abdominal CT scan shows features of chronic calcific pancreatitis with a portal annular pancreas. Dilated MPD (indicated by the blue block arrow) runs posteriorly to the portal vein (indicated by the red chevron arrow). An accessory pancreatic duct (indicated by the yellow block arrow) is noted coursing anteriorly to the portal vein, which is better appreciated on MRCP.

**Figure 13:**
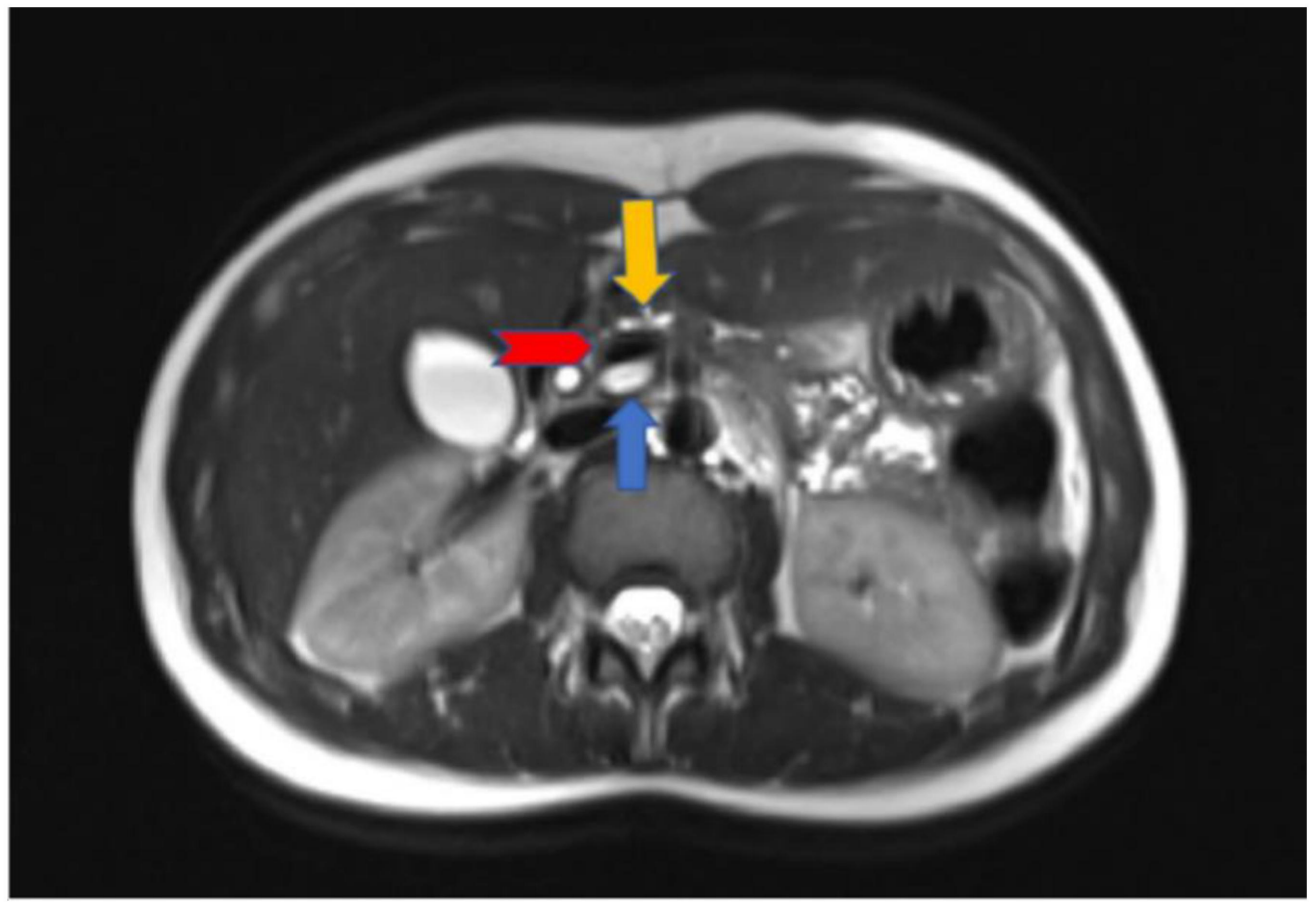
Patient 5: MRCP shows features of chronic calcific pancreatitis with portal annular pancreas. Dilated MPD (indicated by the blue block arrow) runs posteriorly to the portal vein (indicated by the red chevron arrow). An accessory pancreatic duct (indicated by the yellow block arrow) is noted coursing anteriorly to the portal vein.

**Figure 17:**
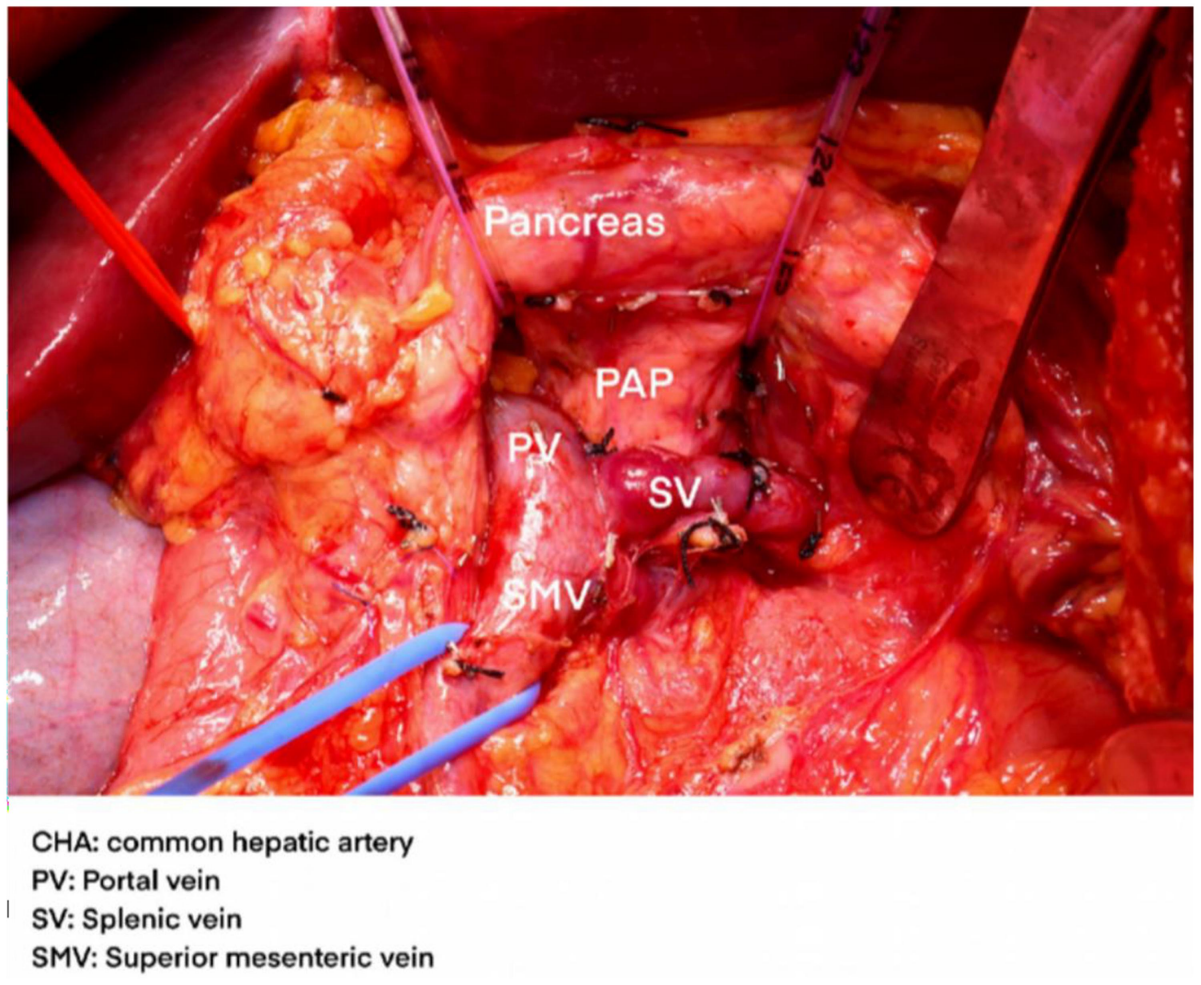
Patient 5: intraoperative photograph—portal annular process before extended resection beyond the fusion point along the second tunnel (mesenteric–coeliac).

**Figure 18:**
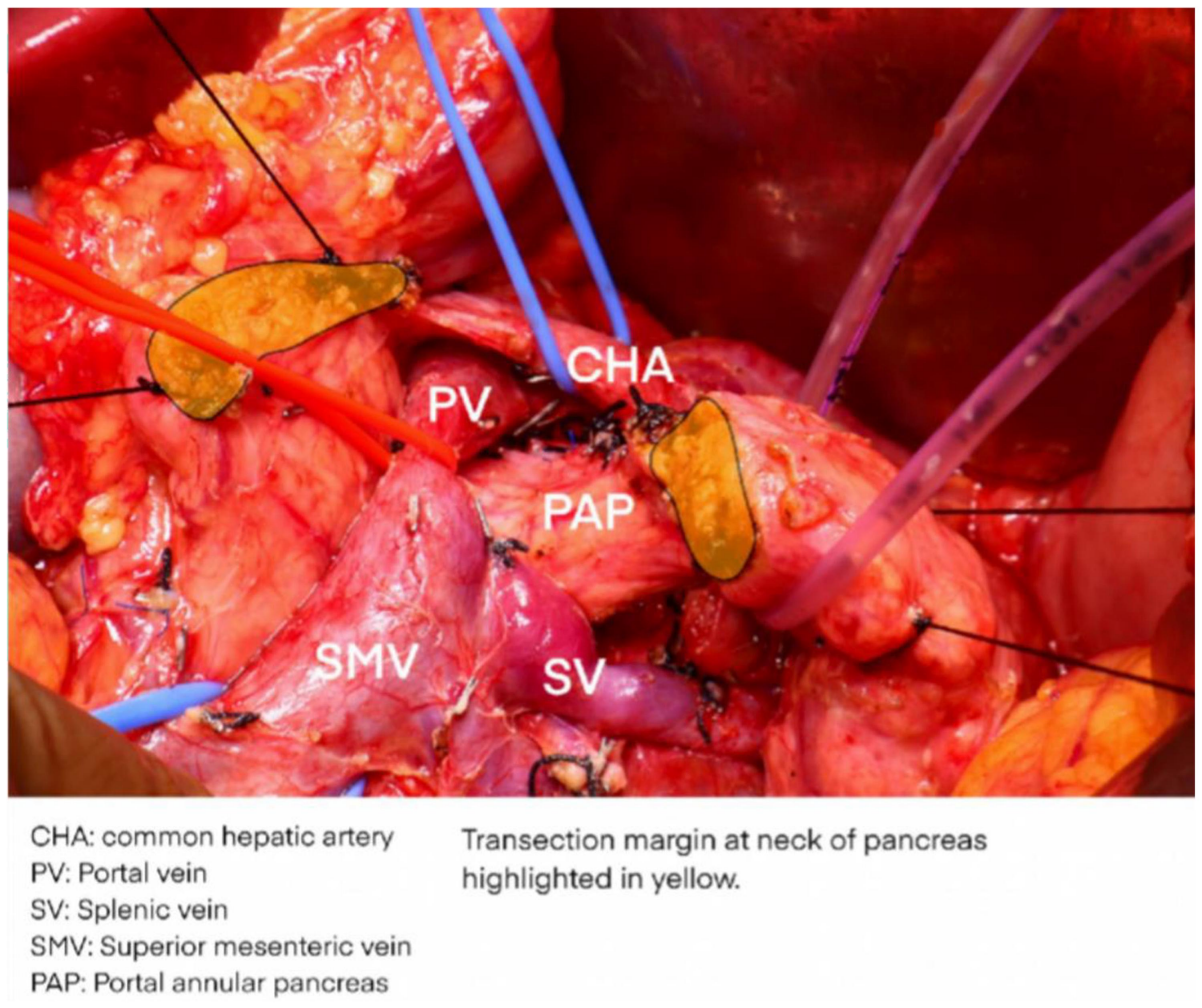
Patient 5: intraoperative photograph—portal annular process after resection of the neck of pancreas to the area of fusion point beyond the second arterial tunnel (mesenteric–coeliac).

**Figure 19:**
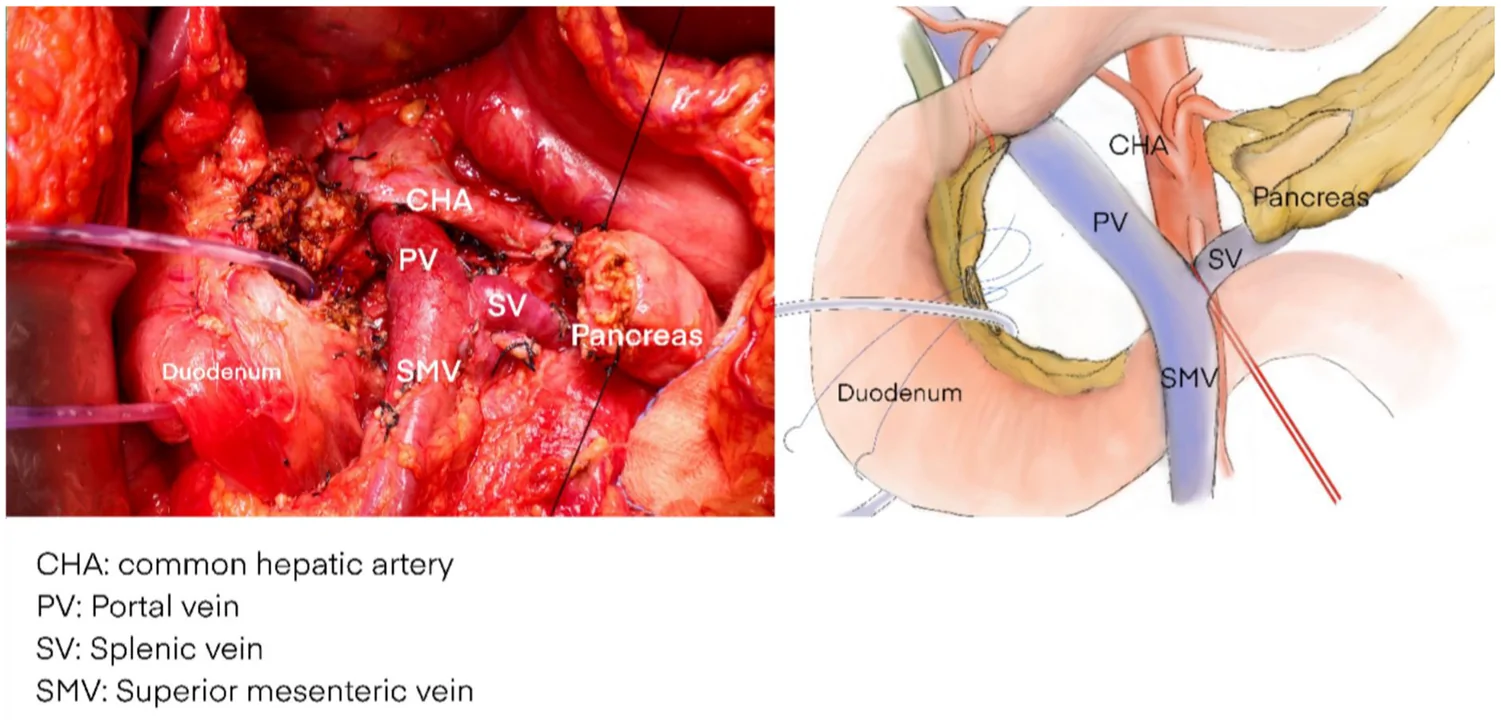
Patient 5: intraoperative photograph—retroperitoneal bed visualized within the c-loop of the duodenum following DPER and the distal stump of the pancreas. CHA, common hepatic artery; PV, portal vein; SV, splenic vein; SMV, superior mesenteric vein.

The drainage procedure after resection was decided based on the stone load. In the patient with obstructive jaundice (Patient 4), the stone load was present only in the head region, and therefore, end-to-side pancreaticojejunostomy was performed. The other patient who had a DPER (Patient 5) and had stones in both the body and tail of the pancreas was planned for a lateral pancreaticojejunostomy. However, the pancreas was not firm. Therefore, the MPD in the remnant stump was partially laid open and a limited pancreaticojejunostomy was done.

In the patients who underwent extended resection, the mean blood loss was 600 mL and the mean duration of operation was 9 h. The mean hospital stay was 14 days. The patient who underwent Frey's procedure had prolonged postoperative ileus, and the one who underwent pancreaticoduodenectomy with extended resection had International Study Group of Pancreatic Surgeries (ISGPS) grade A delayed gastric emptying. The patient who had DPER developed a biochemical leak. None of them had clinically relevant pancreatic fistula (CR-POPF) or postpancreatectomy hemorrhage. All three of them had adequate pain relief with a median visual analog pain score of 3 during a median follow-up of 18 months.

## Discussion

Developmental anomalies of the pancreas are not uncommon in clinical practice. Fusion abnormalities such as pancreas divisum, annular pancreas, and PAP are particularly significant. Many of these do not manifest with symptoms.

Embryologically, the pancreas forms from ventral and dorsal buds arising from the primitive foregut. Normally, during the sixth week of gestation, the ventral bud rotates posteriorly to fuse with the dorsal bud. However, in PAP, this fusion occurs to the left of the superior mesenteric vein and the portal vein, resulting in the uncinate process that wraps around the portal vein or the superior mesenteric vein and joining the dorsal aspect of the pancreatic body (17).

PAP is most often identified incidentally during an operation, indicating that it remains underdiagnosed during preoperative evaluation. At our institution, the prevalence of PAP was 2.5% (18), closely matching the 2.5% reported by Ishigami et al. in CT studies of liver transplant donors (3). Similarly, Harnoss et al. in a systematic review including case reports found a prevalence of 2.4% (2). Other institutional studies have reported lower rates: 1.14% by Karasaki et al. (5) and 0.8% by Yilmaz and Celik (19). A female predominance was also noted, particularly in Karasaki's study (5). Suprasplenic fusion emerged as the most frequent anatomical variant, which is consistent across multiple studies.

Preoperative identification of PAP remains difficult. Harnoss et al. (2) reported that PAP was missed preoperatively in over half (52.9%) of their cases. The presence of PAP makes a significant difference to the operative management in chronic pancreatitis, particularly if the MPD has a retroportal course with significant stone load. This might be one of the reasons for inadequate pain relief following an operation if this is missed. The retroportal MPD, when involved, cannot be drained, and therefore, excision remains the best option. In patients who undergo resection, this can increase CR-POPF if not known preoperatively (20).

The earliest documented case of PAP with chronic pancreatitis was reported by Sugiura et al. (21) in 1987, who described a hypertrophied uncinate process encasing the superior mesenteric vessels in a patient with nodular chronic pancreatitis. Luu et al. (22) later described a patient with both chronic pancreatitis and a branch duct Intraductal papillary mucinous neoplasm (IPMN) in the retroportal pancreatic tissue, which were managed via a pylorus-preserving Pancreaticoduodenectomy (PD). However, to date, no comprehensive case series or systematic review has focused on the management chronic pancreatitis in PAP. Our study is likely the first to focus on the complexity that PAP adds to the management in chronic pancreatitis and to propose an algorithm for this management.

In the presence of obstructive jaundice or head mass, pancreaticoduodenectomy remains the best option. When a pancreaticoduodenectomy is done, a key technical consideration is the management of the pancreatic remnant—specifically distinguishing between the dominant and non-dominant stump. The non-dominant stump, defined as the portal annular process lacking the MPD, may be either stapled or sutured (20). In type 3 PAP with an anteportal MPD, the retroportal portion may be considered for stapling. However, leaving a stapled dominant stump has been associated with significantly higher POPF rates. At our institution, the preferred approach is extended pancreaticoduodenectomy in all these patients, where both dominant and non-dominant stumps are excised. Our experience, supported by recent systematic reviews (23, 24), affirms that extended resection in patients requiring Whipple pancreaticoduodenectomy is associated with the lowest CR-POPF rates—reported at 22.7%. Caution must be exercised when a hepatic artery branch arising from the superior mesenteric artery traverses through the annular segment (23).

When the indication for surgery is pain, both drainage procedures and resection procedures are appropriate options. The choice of surgical procedure is then based on the type of PAP. In type 3 PAP, when the MPD is anteportal, Frey's procedure (Figure 14) will still provide adequate pain relief. However, in type 1/2 PAP, the pain relief will be adequate only if the retroportal annular process bearing the pancreatic duct is excised (Figure 15). This is not achievable with conventional duodenum-preserving pancreatic head resection (DPPHR; Beger procedure). Hence, we recommend a DPER, which involves subtotal excision of the head of the pancreas along with the excision of PAP beyond the splenic artery at the level of fusion (Figure 16).

**Figure 14:**
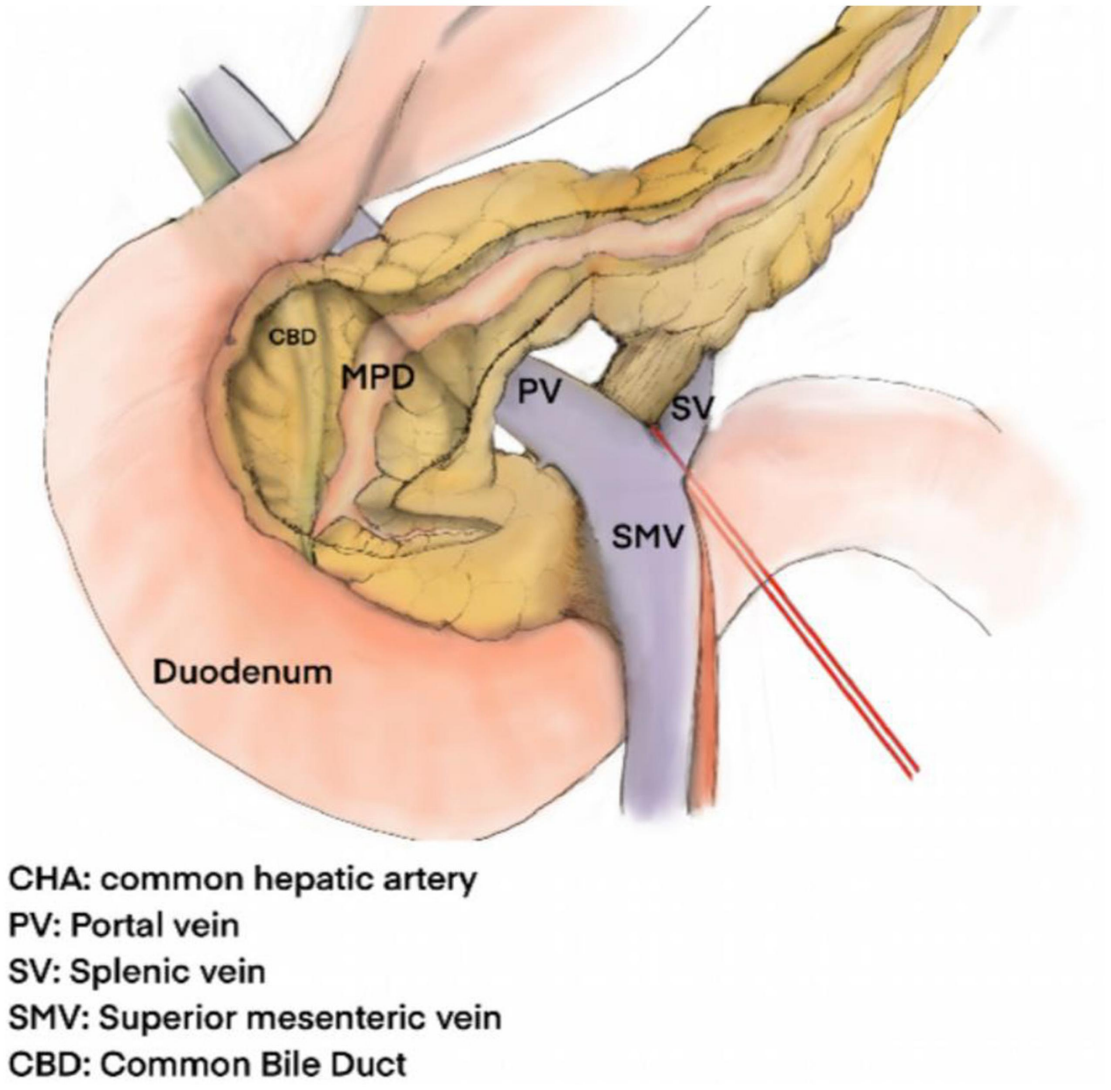
Patient 1: diagrammatic representation of Frey's procedure.

**Figure 15:**
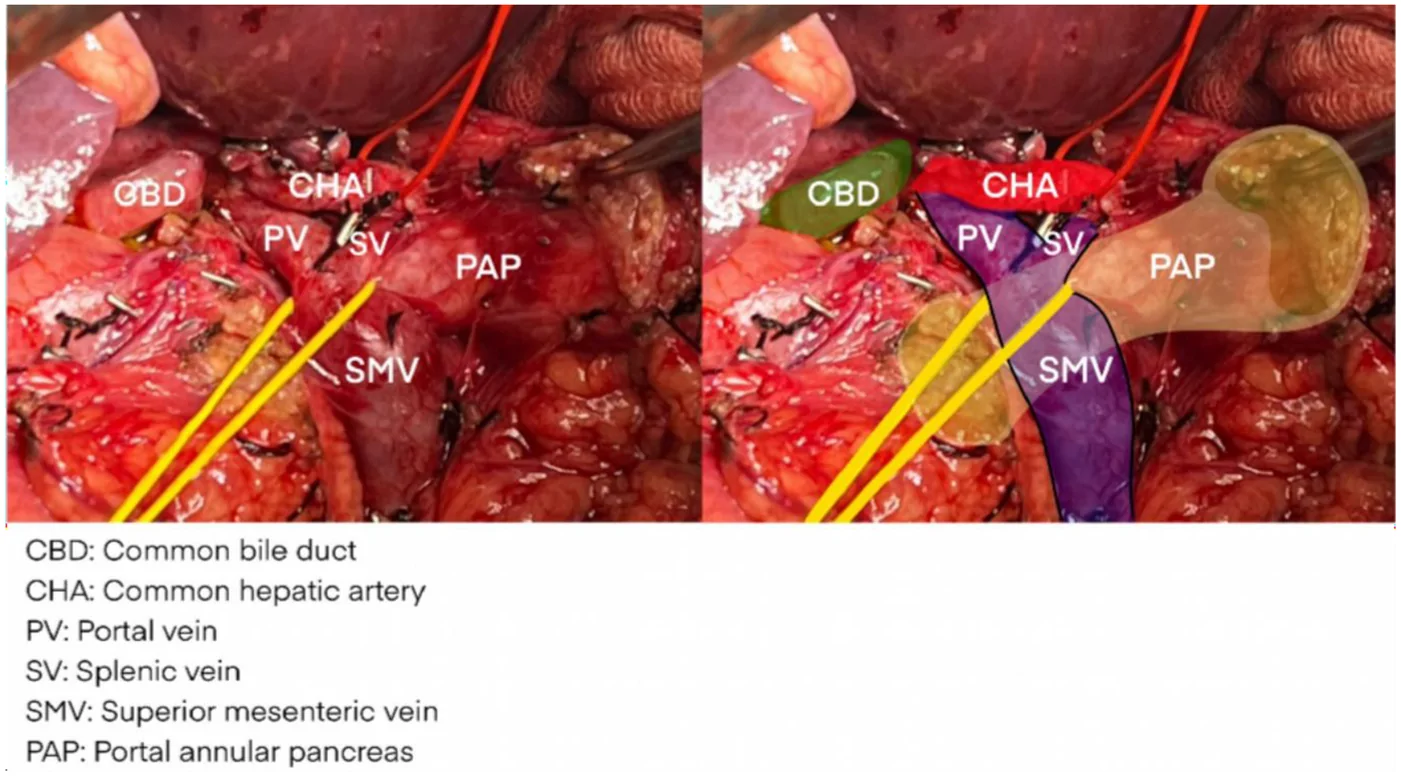
Patient 4: intraoperative photograph—annular process of the pancreas after primary resection at the neck of the pancreas in pancreaticoduodenectomy, demonstrating the area of fusion point beyond the second arterial tunnel (mesenteric–coeliac).

**Figure 16:**
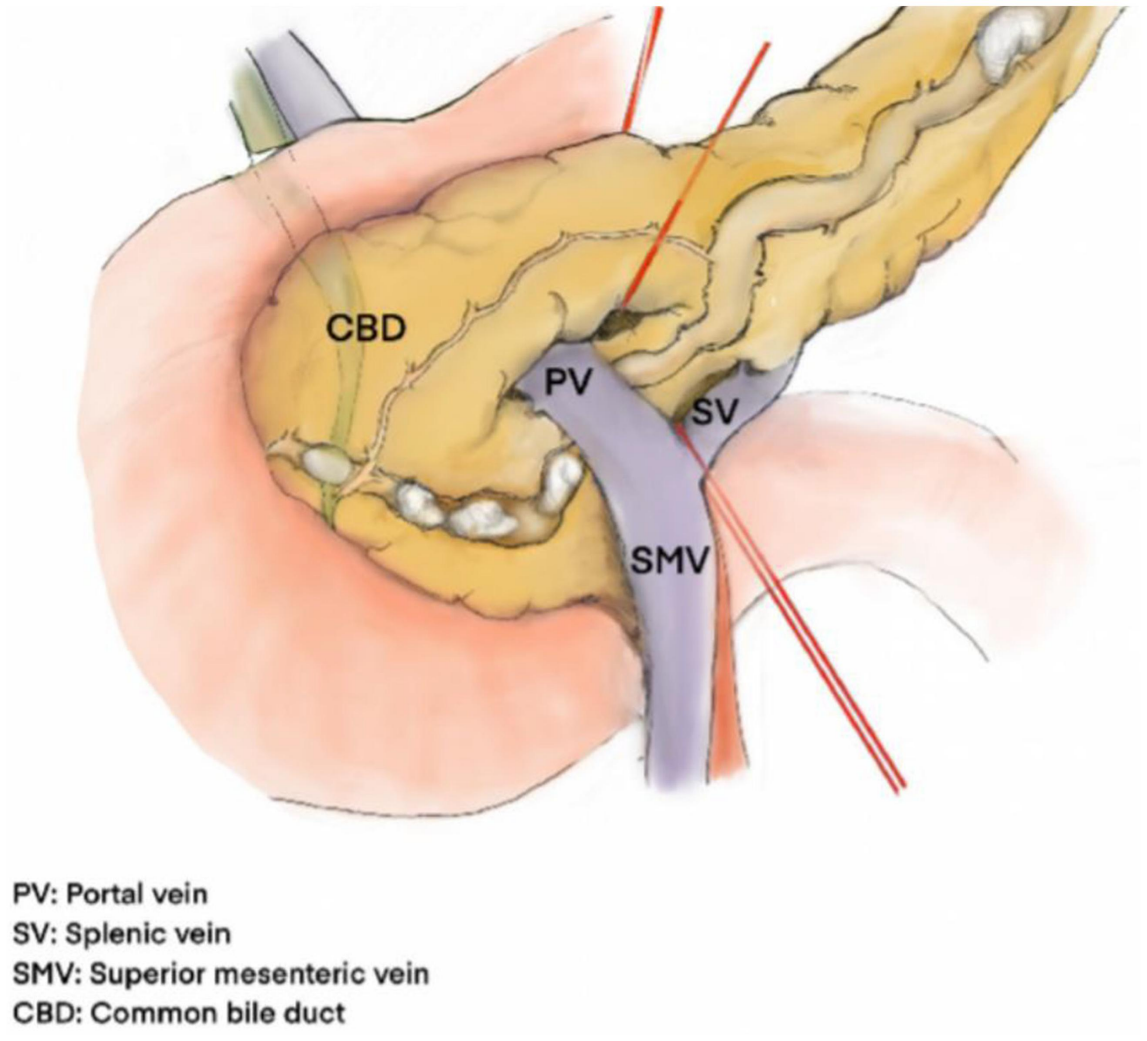
Patient 5: diagrammatic representation of preoperative anatomy.

As a first step, the primary venous tunnel is created (Figure 17), and the neck transection is done at the left border of the portal vein. After creating a secondary arterial tunnel (Figure 18) (mesenteric–coeliac), resection is extended beyond the fusion point to create a unified transection plane for a single duct anastomosis. The head—uncinate process and freed retroportal annular pancreas—is excised near the duodenum, sparing just enough tissue adjacent to the common bile duct and the duodenal rim (Figure 19). The MPD near the ampulla is closed primarily. The retained tissues allow closure of the anterior and posterior pancreatic capsule via interrupted sutures.

Following DPER, the reconstruction is now decided based on the stone load and consistency of the pancreas. An end-to-side pancreaticojejunostomy will suffice if the stone load is only in the head. When the body and the tail also have stones, lateral pancreaticojejunostomy will provide adequate drainage. However, if the consistency of the pancreatic remnant is not firm, limited pancreaticojejunostomy is a safer option.

To the best of our knowledge, there has been no study on the clinical relevance of PAP in the management of chronic pancreatitis. However, our study has limitations such as the small sample size, its retrospective nature, and its single-center setting.

## Conclusions

This study presents the first reported single-center limited case series of chronic pancreatitis associated with PAP. The novel tailored surgical approaches described here were found to be safe and effective, offering promising outcomes in this underrecognized and complex clinical scenario. Further multicenter studies and longer follow-ups are warranted to validate these strategies and establish optimal surgical management for this rare condition.

## Data Availability

The data analyzed in this study is subject to the following licenses/restrictions: image-based data. Requests to access these datasets should be directed to philipjoseph1982@yahoo.com.
